# Hypothalamic hamartomas in adulthood: Clinical spectrum and treatment outcome—A unicenter experience

**DOI:** 10.1002/brb3.1412

**Published:** 2019-10-02

**Authors:** Estefanía Conde Blanco, Carla Anciones Martín, Isabel Manzanares, Francisco Gil López, Pedro Roldán, Antonio Donaire, Jordi Rumiá, Mar Carreño

**Affiliations:** ^1^ Epilepsy Unit Department of Neurology Hospital Clinic Barcelona Spain; ^2^ Department of Neurology Hospital Ruber Internacional Madrid Spain

**Keywords:** adult, epilepsy, hypothalamic hamartoma, radiosurgery, seizures, treatment, vagus nerve stimulation

## Abstract

**Introduction:**

Clinical manifestations of the hypothalamic hamartoma‐epilepsy syndrome (HH‐ES) in adulthood are variable. Efficacy of therapeutic options and outcome are diverse.

**Methods:**

Retrospective study of adult patients diagnosed with a HH in magnetic resonance imaging and epilepsy who attended our tertiary Epilepsy Unit between 2003 and 2018. We report the clinical and electroencephalographic features of a series of adult patients with HH and related epilepsy seen in our center together with the treatments and seizure outcome.

**Results:**

We describe a series of eight patients. Five males (62.5%), median age at evaluation was 28.5 years (IQR: 15.5). Clinical manifestations included focal with preserved and impaired awareness emotional seizures (gelastic seizures [GS]) in six patients (75%), focal tonic, atonic with impaired awareness and focal to bilateral tonic–clonic seizures. Mild GS were the only symptom in one patient. Three patients (37.5%) had endocrinological disturbances such as obesity and hypothyroidism. Fifty percent of the patients showed psychiatric comorbidity such as anxiety disorder and aggressiveness, and two patients had psychomotor delay. Seven patients (87.7%) had drug‐resistant seizures and three of them were treated with radiosurgery. Out of the treated group, only one (33.3%) became seizure‐free 2 years after surgery but developed psychiatric problems. The other two patients had an Engel IV outcome and received a vagal nerve stimulation (VNS) implant. VNS did not lead to changes either in intensity nor in seizure frequency.

**Conclusions:**

Hypothalamic hamartoma‐epilepsy syndrome clinical manifestations in adult patients are as variable as at pediatric age. Outcome of therapeutic options such as radiosurgery or VNS may be poorer at this stage.

## Graphical abstract

Hypothalamic hamartoma‐epilepsy syndrome (HH‐ES) clinical manifestations in adult patients are as variable as at pediatric age. We analyzed adult patients diagnosed with a HH in magnetic resonance imaging and epilepsy who attended our tertiary Epilepsy Unit between 2003 and 2018. Clinical and electroencephalographic features of a series of adult patients with HH and related epilepsy seen in our center together with therapeutic options and outcome are evaluated.

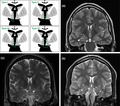

## 1 INTRODUCTION

Hypothalamic hamartomas (HH) are composed of ectopic neural and glial tissue and represent a notable etiology of treatment‐resistant epilepsy. Two different clinical phenotypes are described depending on the location of the hamartoma. Those HH that invade mainly the anterior hypothalamus may develop endocrinological disturbances such as central precocious puberty (CPP) and those HH affecting the posterior hypothalamus and the mammillary bodies associate drug‐resistant seizures that usually start in the early childhood as gelastic seizures (GS) (Harrison, Oatman, & Kerrigan, 2017; Kerrigan et al., 2017).However, HH have a wide clinical expression spectrum (Mullatti et al., 2003; Striano & Striano, 2017) that ranges from a few seizures with normal cognitive development to a catastrophic epileptic encephalopathy (EE) resembling a Lennox‐Gastaut‐like syndrome with cognitive impairment and severe behavioral disturbances (Cross & Spoudeas, 2017; Scheffer et al., 2016). We present the clinical features, electroencephalographic (EEG) recordings, imaging findings, and therapeutic options in a series of adult patients with epilepsy due to hypothalamic hamartoma.

## 2 MATERIAL AND METHODS

We reviewed all patients with epilepsy and HH referred to the Epilepsy Unit of Hospital Clínic (Barcelona, Spain) between 2003 and 2018. We included those patients with history of seizure and magnetic resonance imaging (MRI) evidence of an HH defined by a T2‐weighted image hyperintense and T1‐weighted image hypointense lesion, that involves the hypothalamus, mammillary regions, tuber cinereum, and/or third ventricle floor. Patients with dual pathology were also included.

Evaluation included a detailed clinical history and examination. We analyzed treatment choices, age at evaluation and seizure onset, sex, type of seizures according to the International League Against Epilepsy (ILAE) classification (Delalande & Fohlen, 2003), frequency of seizures, scalp EEG or video‐EEG recordings, MRI findings according to the Delalande and Fohlen classification (Palmini, Paglioli‐Neto, Montes, & Farmer, 2003), endocrine disturbances, psychiatric comorbidities, cognitive status, current antiepileptic drug (AED) therapy, and nonpharmacological treatment of the HH. We report single photon emission computed tomography (SPECT) and subtraction ictal SPECT coregistered to MRI (SISCOM) results, when obtained during admission in patients who went through presurgical evaluation. Nonpharmacological treatment is discussed. The study was approved by the Healthcare Ethics Committee of the Hospital Clínic de Barcelona.

## 3 RESULTS

### 3.1 Demographic and epilepsy history data

Eight patients fulfilled the inclusion criteria, five were males (62.5%), median age at evaluation was 28.5 years (IQR: 15.5) and median age at seizure onset is 2 (range 0.2–13) years of age. Patients had no other risk factors for epilepsy.

Six patients (75%) had focal aware emotive (gelastic) seizures as initial symptom. GS were the only seizure type in one patient who often experienced repetitive gelastic status epilepticus. In the remaining five cases, GS coexisted with other seizure types. One patient (No. 4) referred a mirth sensation or “need of laugh” instead of frank bursts of laugh. Four out of eight (50%) had focal seizures with impaired awareness, three out of eight (37.5%) had focal to bilateral tonic–clonic seizures and one had psychogenic nonepileptic seizures (PNES) in addition to GS. Two patients (No. 3 and No. 8) had a severe EE with several seizure types including GS and focal motor onset with impaired awareness, atonic, tonic, and focal to bilateral tonic–clonic seizures.

Seizure frequency was variable. For example, patient 1 presented monthly 2–3 focal onset impaired awareness seizures with automatisms while patient 6 could have up to 50 GS per day. Clinical features are summarized in Table 1.

**Table 1:** Demographic and epilepsy history data

Patient	Age/Sex	Seizure onset	Types of seizures	Frequency of seizures	EEG recording	Radiological classification
1	47 M	13 years	Focal seizures with consciousness impairment and automatisms	2–3 episodes per month	Video EEG: Interictal: normal background with intermittent bilateral temporal slowing during non‐REM sleep. SW over the right and left frontotemporal regions. Ictal: diffuse electrodecrement followed by spiky beta rhythmic activity on both frontotemporal regions and late delta activity that could focus either on right or left temporal regions	Type III Additional bilateral hippocampal sclerosis
2	47 F	8 years	1. Gelastic seizures 2. Possible PNEs	1. Daily (1–2) 2. Variable	Video EEG: Interictal: normal background. Left mid‐anterior temporal SW. Ictal: polymorphic theta activity over left hemisphere predominant in left frontal region that evolves to theta activity over the left temporal region	Type II
3	19 M	6 months	1. Gelastic seizures 2. Generalized tonic 3. Focal with consciousness impairment 4. Generalized tonic‐clonic	1. Daily: 2–3 per day 2. Monthly: 20–24 per month 3. Daily: 2–3 per day 4. Monthly: 5–6 per month	Video EEG: generalized background slowing with abundant superimposed epileptiform activity. Interictal: frontopolar bilateral spikes. Polyspikes in left hemispheric regions that frequently evolved to generalized spike‐wave complexes. Independent left and right parietal SW. Ictal pattern: bilateral frontotemporal and midline polyspikes in alpha‐beta range that evolved to both frontotemporal regions predominant over the left side in most of the gelastic seizures	Type IV
4	27 M	2 years	1. Gelastic seizures 2. Focal with consciousness impairment	1. Daily: 3 per day 2. Monthly: 1 per week	Video EEG: normal background. Interictal: continuous slowing over the left anterior temporal region. Left anterior temporal SW predominant during NREM sleep. Occasional mid and posterior left temporal SW. Ictal: no clear pattern	Type II Also Right mesial sclerosis. Not diagnosed until EMU admission (age 27)
5	27 M	14 months	1. Gelastic seizures in cluster 2. Generalized tonic‐clonic seizures	1. Daily 2. 1 every 8–9 months	Routine EEG: normal background Interictal: intermittent theta slowing on left temporal region. Ictal: no seizures recorded	Type IV
6	31 F	2 years	1. Gelastic seizures	1. Daily: 50 per day	Video EEG: normal background. Interictal: no epileptiform abnormalities. Ictal: rhythmic generalized activity maximum over both frontal regions that was seen within the first 15 s after the seizure onset	Type II
7	30 F	5 years	1. Focal onset seizures with impaired consciousness 2. Generalized tonic‐clonic	1. One every 2 weeks 2. One every 2–3 years	Routine EEG: Normal background. No epileptiform activity recorded. No seizures recorded	Type III
8	20 M	2 months	1. Gelastic seizures 2. Tonic seizures 3. Atonic seizures	1. Daily: 6–7 per day 2. Weekly: 1 every 2–3 days 3. Daily: 1 per day	Video EEG: normal background. Interictal: no epileptiform abnormalities. Ictal: diffuse electrical electrodecrement with low voltage intercalated paroxysmal fast activity. Occasionally, seizures started with a high delta wave more prominent either over the right or left hemispheres	Type II

Abbreviation: EEG, Electroencephalography; F, Female; M, Male; SW, Sharp waves.

### 3.2 Neuroimaging

#### 3.2.1 MRI

**Figure 1:**
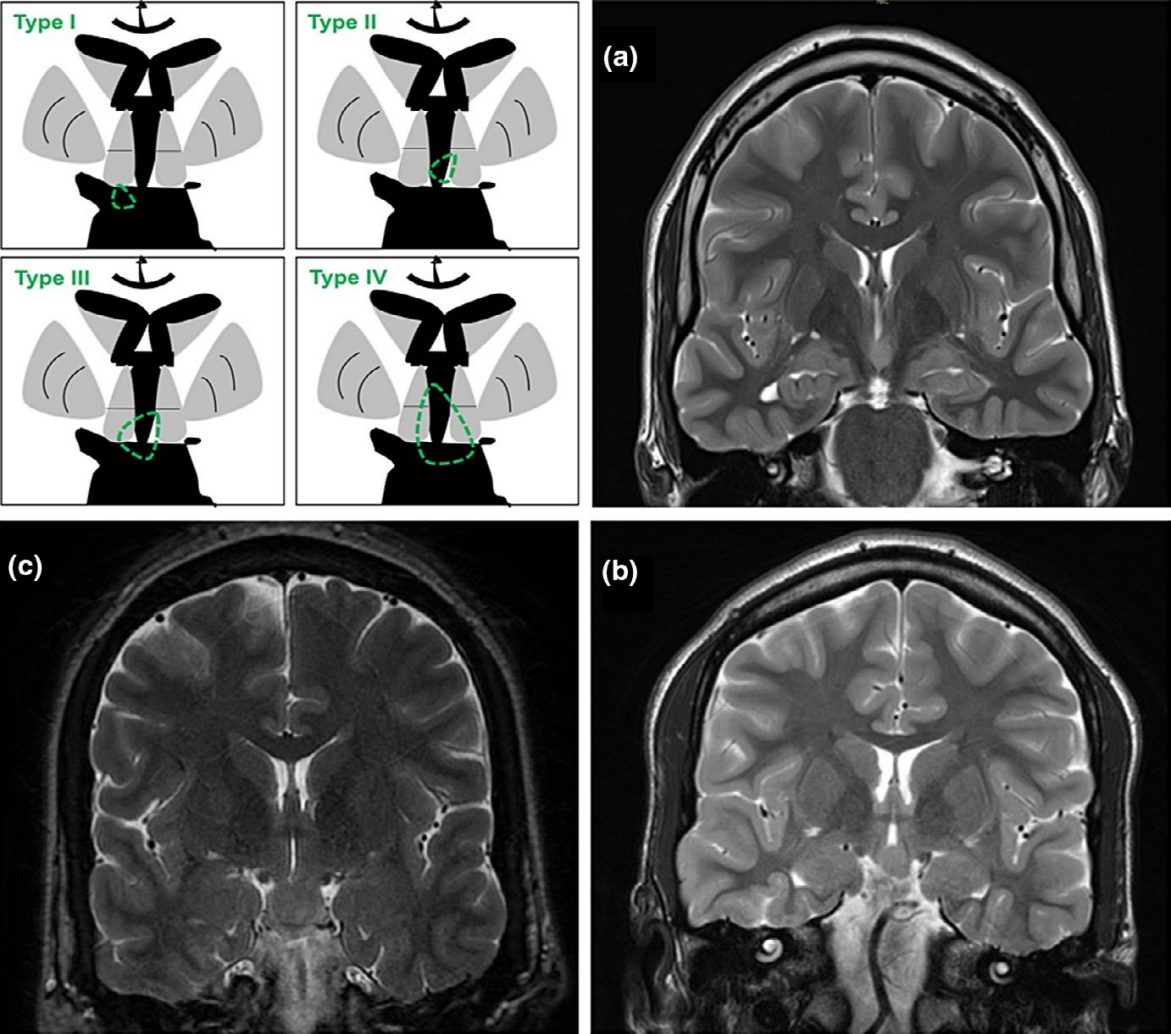
Neuroimage. (a) MRI slice in the coronal plane using T2 weighted image from patient No. 2. An intrahypothalamic hamartoma lesion is attached to the left lateral wall of the third ventricle and completely above the floor of the third ventricle (type II). This patient also had left side hippocampal sclerosis (dual pathology). (b) Same sequence belonging to patient No. 8. There is a HH arising from the left wall of the third ventricle that extends beyond its inferior limit (Type III). (c) Patient No. 5 with a giant lesion emerging from the inferior walls of the third ventricle (Type IV)

All patients had lesions compatible with HH in 1.5 and 3 Tesla Brain MRI performed with an epilepsy protocol. Median diameter size was 13 mm (IQR: 5.585). Four patients had Type II hamartomas (vertical plane of attachment to the wall of the third ventricle completely above the normal position of the floor of the third ventricle), two of them had Type III hamartomas (the plane of attachment extends both above and below the normal position of the floor of the third ventricle), and two of them had Type IV lesions or “giant” hamartomas. None had Type I lesions (pedunculated with a horizontal base of attachment below the normal position of the third ventricle). One patient showed unilateral hippocampal sclerosis (HS) and another patient had bilateral HS. See Figure 1.

#### 3.2.2 Functional neuroimaging studies

Four patients underwent ictal and interictal SPECT with further SISCOM analysis. Patient No. 6 showed nonconclusive findings. Patient No. 1 had right temporal hyperperfusion extending to the ipsilateral insula and to the hamartoma. Patient No. 2 showed left temporal hyperperfusion predominant over the anterior pole. Patient No. 8 had left occipital ictal hyperperfusion during a GS, not consistent with the ictal electroencephalographic recordings. See Table 2.

**Table 2:** Functional imaging, comorbidities, and treatment

Patient	Ictal SPECT/SISCOM	Endocrine comorbidities	Development/Psychiatric comorbidities	AED therapy	Non pharmacological treatment	Other
1	Right temporal hyperperfusion extending to ipsilateral insula and toward the HH	None	None	CBZ, LTG, CLB	No	Tried VPA, LGT, PHT, LCM without efficacy. SEEG implantation including hamartoma offered
2	Ictal SPECT: left temporal hyperperfusion, predominant over anterior pole	None	Anxiety disorder	PB, TPM, ESL	No	PNES as disabling as GS
3	Not performed	Central obesity and hyperlipidemia	Severe encephalopathy with severe developmental delay and behavioral problems	CBZ, CLB, LCM, ZNS	Radiosurgery at age of 6 years VNS at 16. Switched off because of lack of efficacy and development of apneas	Tried OXC, VPA, TPM, LEV, VG, PHT and ketogenic diet. New radiosurgery being considered
4	Not performed	Hypothyroidism	None	No treatment	No	Tried LCM, TPM, VPA, LTG, ZNS, withdrawn because of for side effects
5	Not performed	None	None	OXC, LEV, TPM	No	Tried GBP
6	Nonconclusive: no evidence of areas of hyperperfusion in ictal SPECT. Negative SISCOM	None	Depression and anxiety disorder Psychotic episodes (associated to AEDs)	OXC, LCM, CLZ	Linear accelerator stereotactic radiosurgery at age of 27. Seizure‐free after surgery but disabling psychiatric disturbances	Tried LEV, TPM, VGB, TGB, CLB
7	Not performed	None	None	LGT	No	Tried CLZ
8	Ictal hyperperfusion in the left occipital lobe	Hypothyroidism	Severe memory impairment, psychiatric disturbances in childhood with attention deficit and aggressiveness	CBZ, TPM, LCM, BRV	Radiosurgery at 15 years (ineffective) VNS at 20 years	Tried VPA and PER

Abbreviations: AED, Antiepileptic drugs; BRV, Brivaracetam; CBZ, Carbamazepine; CLB, Clobazam; CLZ, Clonazepam; ESL, Eslicarbazepine; GBP, Gabapentin; GS, gelastic seizure; HH, Hypothalamic hamartoma; LCM, Lacosamide; LEV, Levetiracetam; LGT, Lamotrigine; OXC, Oxcarbazepine; PB, Phenobarbital; PER, Perampanel; PHT, Phenytoin; TGB, Tiagabine; TPM, Topiramate; VGB, Vigabatrin; VNS, Vagus Nerve Stimulation; VPA, Valproic acid.

### 3.3 EEG data

Electroencephalographic findings in our group are summarized in Table 1. Six of the patients underwent prolonged video‐electroencephalogram (EEG) recording and two of the patients underwent routine EEG. Five patients showed interictal epileptiform activity over the temporal lobe, either bilateral or unilateral. Patient No. 3, diagnosed of an epileptic encephalopathy, had frequent bilateral frontopolar spikes, polyspikes, and spike and wave complexes predominantly over the left hemisphere and independent left and right parietal sharp waves. GS were recorded in six patients. Two patients had a distinct ictal pattern named as evolving generalized fast rhythmic activity (EGFRA), first reported by Mullati et al. which consists of an abrupt background diffuse attenuation followed by a brief burst of fast generalized polyspikes and then bursts of generalized rhythmic fast activity lasting a few seconds, at a variable frequency between 14 and 24 Hz, which then evolved into slow spike–wave activity. This pattern was the most consistent one through the sample.

### 3.4 Outcome and comorbidities

None of our patients have had signs of CPP; two had hypothyroidism and were on hormonal therapy; one had obesity and dyslipidemia. Six patients (75%) had a normal neurodevelopment, one had anxiety, and another had an isolated episode of psychosis which was attributed to antiepileptic therapy. The remaining two patients (25%) had severe cognitive impairment: one of them developed a severe encephalopathy with language disturbance, aggressiveness and a compulsive eating disorder; the other patient also had severe behavioral alterations with aggressiveness and a severe verbal memory loss corroborated by neuropsychological studies. Both patients began with GS before 1 year of age.

### 3.5 Treatment

Median number of AEDs per patient was three drugs (range 0–4). Two patients took <3 AEDs: one of them had mild occasional GS and decided to stop medication on his own; the other patient was controlled with one drug (Table 2). Three patients (37.5%) underwent surgery: one patient was treated with Gamma Knife (GK) at the age of 6, in a foreign country, with no significant clinical improvement and underwent GK again at 19 years of age. The other two patients received linear accelerator stereotactic radiosurgery (LINAC) of the HH at 27 and 15 years, respectively (gamma knife is not available in our region). One patient achieved Engel Ib and had complete cessation of GS 2 years after procedure; the 1‐year post‐LINAC MRI displayed contrast enhancement and edema in tuber cinereum‐mammillary bodies‐interpeduncular cistern region. The other two patients had an Engel IV outcome. The patient who initially had treatment with GK and was retreated showed a subtle improvement in intensity the first 2 months afterward and progressively fell back into the previous frequency and duration.

In our series, no severe side effects occurred, such as permanent amnesic and endocrinologic complications, which were reported with other surgical techniques. One of the patients experienced aggravation of anxiety, emotional lability, menstrual irregularities, and compulsive eating disorder but hypothalamic function was normal. This was the patient who displayed edema in the MRI and became seizure‐free. Neuropsychological follow‐up assessments did not show a consistent neurocognitive deficit.

The other patients showed no changes in seizure frequency after the procedure. The patient who achieved an Engel Ib outcome received radiosurgery at an older age compared with the other two patients. Median epilepsy duration at radiosurgery was 14.83 years (range 5.5–25 years), but age at onset did not seem to be a determinant factor contributing to outcome in our series.

Two out of the three patients treated with radiosurgery and poor outcome underwent vagus nerve stimulation (VNS) implantation. In the first case, it had to be switched off because of lack of efficacy and respiratory side effects (apneas and hypopneas). In the second case, the effect of the device was quite modest and did not achieve seizure control. Initially output current parameters oscillated from 1 to 2.5 mA, signal frequency 30 Hz, signal pulse width 500 us, and “on” and “off” time: 30 s on and 5 min off.

## 4 DISCUSSION

Our series is a good example of the variable clinical expression of HH in the adult age and its low prevalence. HH is a rare condition, with an estimated prevalence of 1–2 cases per 100,000 population (Striano et al., 2012). We practice at a referral center in Spain for drug‐resistant epilepsy, and we identified eight patients in our database. We could not find any patients with frank GS in the context of frontal or temporal lobe epilepsy, without associated HH. HH is usually sporadic, but in 5% of cases can be associated with the Pallister‐Hall syndrome (Georgakoulias, Vize, Jenkins, & Singounas, 1998; Scheffer et al., 2016). The initial presentation in the sporadic cases usually occurs in the first months of life as GS. In 50% of the cases, it may progress to a disabling epileptic syndrome with multiple types of seizures typically refractory to antiepileptic therapy resembling a severe encephalopathy with possible additional features such as endocrinological and psychiatric disturbances (Kerrigan et al., 2017; Ng et al., 2006; Oehl et al., 2010; Striano et al., 2012).

Our series also illustrates that diagnosis may be challenging specially in the most benign cases. Patient No. 4 had very short focal seizures with consciousness impairment and mild GS, that he defined as mirth or “pressure to laugh” and which had a minor impact in his quality of life. He was diagnosed of HH in our Unit, 25 years after seizure onset at 27 years of age. This case represents a mild end to the clinical spectrum and reminds us of the fact that cognitive or behavioral disturbances may not always be present as previously stated (Striano et al., 2002).

Children with HH‐epilepsy usually have no cognitive impairment prior epilepsy onset; however, after seizures begin, especially if this happens at an early age, patients develop progressively behavioral deterioration and developmental delay, such as oppositional defiant disorder (83%), attention deficit/hyperactivity (75%), aggression (89%), behavioral disorder (33%), speech disorder/learning impairment (33%) and anxiety and mood disorders (17%) (Cross & Spoudeas, 2017). The deterioration seems more obvious in those patients who develop an EE and not only GS. Patients without cognitive and behavioral deterioration present minor attacks, mild GS and often smaller lesions that did not protrude into the lumen of the third ventricle (Striano, Striano, Sarappa, & Boccella, 2005).

Most of the patients in our series had 1 or 2 seizure types starting at the age of 2 years or afterward, presented type II HH in MRI and had no psychiatric disorders. Patients with the most severe behavioral alterations and neurodevelopmental delay (patients No. 3 and No. 8) presented daily, different seizure types since early childhood and displayed lesions invading the third ventricle. Size was variable and patients with longer epilepsy duration had smaller median size than those with less duration of the disease with a negative correlation coefficient (*r*: −.4751) not statistically significant (*p*: .19) due to the low sample number.

There is not a definitive predictor of evolution patterns and clinical variability in HH patients but it is known that clinical expression and syndrome severity are related to the dimension, location, and attachment of the HH (Georgakoulias et al., 1998). Often the sessile masses associate epilepsy and the pedunculated ones may be related to endocrinological disturbances and/or to visual impairment (Kameyama, Masuda, & Murakami, 2010). Some authors suggest that larger hamartomas are frequently associated with higher seizure severity, cognitive impairment, and behavioral disorder as signs of EE probably due to hypothalamic‐thalamic‐mammillary connections dysfunction (Deonna & Ziegler, 2000; Frattali et al., 2001; Prigatano, 2007; Tezer, Pektezel, Gocmen, & Saygi, 2014). In our experience, the most severe cases with pronounced cognitive decline were the ones that started earlier with GS, suggesting that age of onset may also play a predictor role in HH‐ES outcome. Severity may be also related to duration of epilepsy, the cases who started earlier and did not achieve seizure freedom may progress to an EE. However, patient No. 5 presented a giant hamartoma and early onset of GS but showed no developmental delay, behavioral or psychiatric problems. This raises the question of whether location and invasion of close structures may be the most important factors related to epilepsy severity.

The hamartoma location influences the regulation of hormone release. Pedunculated HH occupying the anterior hypothalamus (Type I) may cause endocrinological disturbances, being the CPP the most frequent one, probably because of a premature pulsatile release of gonadotropin‐releasing hormone (GnRH) from the hypothalamus (Harrison et al., 2017; Striano & Striano, 2017). CPP and epilepsy coexist in up to 45% of the patients undergoing surgical treatment for epilepsy (Oehl et al., 2010). Other disturbances include secondary or tertiary hypothyroidism and weight gain with obesity. Infrequent comorbidities are Addisonian crisis and central diabetes. Sleep disturbances like hypersomnia (Dunn, Deroo, & Rivara, 2001) and reduction in REM sleep have been described and may worsen seizure severity (Cross & Spoudeas, 2017; Troester et al., 2011). None of the patients in our series had history of CPP, even when masses were large enough to invade the anterior hypothalamus. Two patients presented hypothyroidism and another one severe central obesity and dyslipidemia.

The EEG monitoring shows a wide disparity too. Routine (interictal) recordings range from normal EEG results to multiple interictal epileptiform features demonstrating generalized, multifocal, and/or focal discharges and suggesting that EEG is not a specific test for the diagnosis of HH‐epilepsy disorders (Scholly et al., 2013). Ictal recordings may be variable too, though GS show no obvious ictal changes in the majority of events (Gutierrez et al., 2016), non‐GS may show epileptic activity mainly in frontal and temporal lobes (García‐Morales et al., 2007; Scholly et al., 2013).

This lack of electric correlate, especially in patients with subtle GS, makes the diagnosis of HH‐ES challenging in patients with less severe seizures. Scholly et al. introduced the concept of “hypothalamic plus” epilepsy, as a result of secondary epileptogenesis mechanisms referring to the functional involvement of the frontal and temporal lobes in the HH epileptogenic network (Kerrigan, Ng, Chung, & Rekate, 2005; Régis et al., 2017; Striano & Striano, 2017). In our study, one patient showed unilateral HS and another patient had bilateral HS. This findings may relate to potential secondary epileptogenesis which is probably linked to the anatomic and functional connectivity of the hypothalamus with frontal lobes, limbic system, and thalamus (Wu, Gao, Shen, Qiu, & Kerrigan, 2015). HH has been demonstrated to be an intrinsically epileptogenic lesion from stereoelectroencephalography recordings within the lesion (Kahane, Ryvlin, Hoffmann, Minotti, & Benabid, 2003; Munari et al., 1995). Kahane et al. proposed a pathway based on the HH strong connectivity to mammillo‐thalamo‐cingulate tract that could spread epileptic activity to the cingulate and from here to temporal or frontal areas. A secondary “kindling”‐like effect of the primary HH could explain why these interconnected brain areas develop the ability to independently generate seizures (Harvey & Freeman, 2007; Kahane et al., 2003).

Cellular mechanisms responsible for epileptogenesis are unknown but the intrinsic epileptogenicity of HH was explained by the microanatomic organization and electrophysiologic characteristics of HH neurons. The predominant type of neurons are small HH neurons (SHH), they account for approximately 90% of the cells and they have a tendency to cluster (Fenoglio et al., 2007). These SHH appear to have an interneuron‐like phenotype that expresses γ‐aminobutyric acid (GABA) and a tendency for intrinsic pacemaker‐like firing activity. There are also large HH (LHH) neurons which are functionally immature and usually at rest but with paradoxical depolarization and firing in response to GABA ligands. LHH represent around 10% of the neuron population in HH tissue and seem to have a phenotype of excitatory projection‐type neuron by using excitatory glutamate as their primary neurotransmitter (Striano et al., 2002).

Treatment should be individualized depending on the radiological characteristics of the HH, AED response and tolerability, seizure severity, and availability of surgical techniques in each center. Epilepsy associated with HH is known to be in most cases drug‐resistant and there is no data whether medication makes any difference to the natural history of the condition or any longer‐term benefit (Scheffer et al., 2016). Most series however come from surgical centers and this may introduce a significant bias toward more severe cases.

Surgical treatment may have an effect on seizures and additional comorbidities producing a “running‐down‐like” phenomenon, stopping or even reverting the evolution of the HH‐ES and should be performed as early as possible (García‐Morales et al., 2007; Régis et al., 2017; Savard, Bhanji, Dubeau, Andermann, & Sadikot, 2003; Scholly et al., 2013; Striano & Striano, 2017). There is a variety of classical surgical techniques to treat HH including the endoscopic approach such as pterional, orbitozygomatic, or transcallosal interforniceal. However, endocrinological toxicity (malignant obesity, diabetes insipidus, etc.) and neuropsychological deterioration have been reported with these techniques. Radiosurgery with Gamma Knife (GK) was found to be a safe and effective option in the treatment of HH‐ES (Castinetti, Brue, Morange, Carron, & Régis, 2017). Régis et al. published a prospective series of 57 patients with HH treated with GK with a 3‐year follow‐up in 48 of them and a range of age from 3 to 50 years. Good result (Engel class I or II) was achieved in 68.8% of the patients in terms of seizure control and none of the patients showed cognitive decline or disabling endocrinological deficits after treatment (Burrows et al., 2016). The latest surgical approach for HH associated with refractory epilepsy is the magnetic resonance imaging‐guided laser interstitial thermal therapy (MRg‐LITT), which offers the advantage of real‐time imaging of direct thermal ablation with minimal major surgical complications (Du, Gandhi, Rekate, & Mehta, 2017).

In our sample, only patient 6 was completely seizure‐free 2 years after an elective LINAC procedure. This patient had a severe worsening of psychiatric symptoms which was attributed to a possible “forced normalization” process. The patient had a previous history of 2 AEDs‐related psychotic episodes, after trying Topiramate and Levetiracetam, respectively. No memory decline was observed 6 years after radiosurgery but the patient still has many obsessive thoughts and excoriation disorder with compulsions of skin scratching. The two remaining patients treated with GK and LINAC did not show either a regression or improvement of the clinical symptoms induced by the HH. In our small series, radiosurgery induced necrosis was associated to a better seizure outcome, although no MRI data from the first surgery was available in patient number 3, who was operated in another country during childhood at 6 years of age. The variability of results after radiosurgery may be attributed to several factors. Some studies postulate complete disconnection from mammillary bodies as the target to achieve seizure freedom, while others suggest that disconnection may not be a good option and the lesion should be entirely targeted. The fact that two of the patients displayed no response to this therapeutic option raises the point of the importance of an early treatment.

Only small studies of HH and VNS therapy have been published suggesting that this device could have a very modest effect on seizure control (Brandberg, Raininko, & Eeg‐Olofsson, 2004; Murphy, Wheless, & Schmoll, 2000). We implanted the VNS device in two patients with intractable epilepsy who previously failed to respond to radiosurgery. Output current parameters were modified following absence of response to different duty cycles. In our sample, there was no effect on seizure frequency or severity, and only one of them experienced behavior improvement. As it is known for other therapies, late use of drugs and devices is associated to a worse seizure outcome, due to increased drug resistance.

Hypothalamic hamartomas diagnosed in adulthood may show a poorer prognosis to surgical treatment than during childhood because comorbidity and encephalopathy tend to remain following the intervention. In our sample, out of three patients who began seizures in the first months of life, two progress to a disabling epileptic syndrome with multiple types of seizures refractory to antiepileptic therapy, resembling a severe encephalopathy, with additional cognitive and psychiatric disturbances. Lack of seizure control and impairment of mental development have a direct influence on social adaptation and usually condemn patients to depend on a caregiver. Patients with HH‐ES develop progressively behavioral deterioration and developmental delay. However, there are some patients that may present with early seizure onset but represent a mild end to the clinical spectrum as illustrated by patient No. 4. This fact reminds us that cognitive or behavioral disturbances may not always be present and leads our current of thought to the point that location and connectivity of the HH to close structures may be the most important factors related to epilepsy severity.

## 5 CONCLUSION

Hypothalamic hamartoma‐epilepsy syndrome is a rare condition. In adult age, it includes a wide variety of clinical, radiological and EEG findings. Clinical manifestations may be influenced by the location of the lesion and age of seizure onset. Diagnosis may be difficult in some patients with subtle seizures and normal cognition. Treatment options should be considered on an individual basis. Pharmacological and nonpharmacological options in our series of adult patients with drug‐resistant epilepsy related to HH often had a poor outcome with psychiatric complications.

## CONFLICT OF INTEREST

None of the authors have nothing to disclose.

## Data Availability

The data that support the findings of this study are available on request from the corresponding author. The data are not publicly available due to privacy or ethical restrictions.
